# The diagnostic value of electrocardiogram-based machine learning in long QT syndrome: a systematic review and meta-analysis

**DOI:** 10.3389/fcvm.2023.1172451

**Published:** 2023-06-07

**Authors:** Min-Juan Wu, Wen-Qin Wang, Wei Zhang, Jun-Hua Li, Xing-Wei Zhang

**Affiliations:** ^1^ School of Nursing, Hangzhou Medical College, Hangzhou, China; ^2^ School of Public Health, Hangzhou Normal University, Hangzhou, China; ^3^ School of Nursing, Hangzhou Normal University, Hangzhou, China; ^4^ School of Clinical Medicine, Hangzhou Normal University, Hangzhou, China

**Keywords:** machine learning, ECG, LQTS, systematic review, meta-analysis

## Abstract

**Introduction:**

To perform a meta-analysis to discover the performance of ML algorithms in identifying Congenital long QT syndrome (LQTS).

**Methods:**

The searched databases included Cochrane, EMBASE, Web of Science, and PubMed. Our study considered all English-language studies that reported the detection of LQTS using ML algorithms. Quality was assessed using QUADAS-2 and QUADAS-AI tools. The bivariate mixed effects models were used in our study. Based on genotype data for LQTS, we performed a subgroup analysis.

**Results:**

Out of 536 studies, 8 met all inclusion criteria. The pooled area under the receiving operating curve (SAUROC) for detecting LQTS was 0.95 (95% CI: 0.31–1.00); sensitivity was 0.87 (95% CI: 0.83–0.90), and specificity was 0.91 (95% CI: 0.88–0.93). Additionally, diagnostic odd ratio (DOR) was 65 (95% CI: 39–109). The positive likelihood ratio (PLR) was 9.3 (95% CI: 7.0–12.3) and the negative likelihood ratio (NLR) was 0.14 (95% CI: 0.11–0.20), with very low heterogeneity (*I*^2 ^= 16%).

**Discussion:**

We found that machine learning can be used to detect features of rare cardiovascular disease like LQTS, thus increasing our understanding of intelligent interpretation of ECG. To improve ML performance in the classification of LQTS subtypes, further research is required.

**Systematic Review Registration:**

identifier PROSPERO CRD42022360122.

## Introduction

Congenital long QT syndrome (LQTS) is characterized by a prolonged QT interval and abnormal T wave morphology on the electrocardiogram (ECG) (1, 2). Clinically, patients are often asymptomatic, but can suffer from syncope, seizures, or sudden cardiac death (3, 4).

A prevalence of 1:2,000–3,000 is estimated to exist for LQTS, which represents a very rare condition (2, 5, 6). A main method for diagnosing LQTS is to measure the heart rate-corrected QT interval (QTc) (2, 4). Specific ECG features, such as T-wave morphologies, QT interval changes on treadmill QT-stress or epinephrine tests, that may help to diagnose LQTS, but may not be reliable enough to provide accurate diagnostic value (7–11). Even though LQTS is characterized by QT prolongation, many patients appear normal on the ECG, despite they were clinically and/or genetically diagnosed (12, 13). Consequently, diagnosing LQTS is particularly challenging for general cardiologists, resulting in this disease being significantly underdiagnosed (4, 14).

Many studies have demonstrated that physicians have difficulty reading ECGs in clinical situations (15, 16). A number of researchers have used machine learning (ML) to investigate whether artificial intelligence (AI) can enhance ECG interpretation and clinical decision-making. ML can potentially take into account subtleties in ECG waves that humans do not consider in routine interpretations of ECG signals. It has been shown that ML improves ECG interpretation and clinical decision-making for several common cardiovascular diseases like atrial fibrillation or heart failure (17). Several high-quality studies have been conducted on ML algorithms for LQTS, but their diagnostic value remains unclear (18–21). Detecting and identifying LQTS properly is vital to preventing syncope, seizures, and sudden cardiac death in LQTS people. Consequently, a meta-analysis was conducted in order to discover whether ML algorithms could be used to identify LQTS.

## Materials and methods

This study was performed according to the PRISMA statement (22) and was registered in PROSPERO (CRD42022360122).

### Search strategy

The Cochrane, EMBASE, Web of Science, and PubMed databases were searched from inception through 16 August 2022. Supplementary Table S1 provided a detailed description of the search strategy. The references of all the studies included in this study were also manually searched.

### Criteria for inclusion and exclusion

Abstracts and titles of the retrieved studies were reviewed by two authors. Study eligibility criteria were as follows: (1) clearly described ML models and ECG regions used in the LQTS detection, (2) presented the results of the ML algorithms and LQTS detection, (3) be written in English, (4) patients with complete ECG records, (5) provided sensitivity and specificity information, (6) provided the number of LQTS patients, (7) explicitly described data sources and datasets used. These were the criteria for exclusion: (1) only reported risk factors and incidence of LQTS, (2) studies with incomplete data, (3) studies not in English. There was no restriction on the region or year of publication.

### Definition of LQTS

According to HRS/EHRA/APHRS Expert Consensus Statement Recommendations (23), a diagnosis of LQTS is made in the presence of QTc ≥ 500 milliseconds (ms) in repeated 12-lead ECGs, or in the presence of a QTc between 480 and 499 ms in a patient with unexplained syncope, or in the presence of the Schwartz score ≥ 3.5 points. A secondary cause for QT prolongation should be ruled out in all of these cases. Additionally, LQTS can also be diagnosed if one of the LQTS genes carries an unequivocally pathogenic mutation. It is estimated that 90 percent of genotype-positive patients and 75 percent of all clinically diagnosed LQTS cases are caused by three main genes. LQT1 and 2 are derived from functional loss-of-function variants in the potassium channel genes KCNQ1 and KCNH2, respectively. LQT3 is based on gain-of-function variants in SCN5A (2). These genes respectively encode for the slow and rapid delayed rectifier current and the fast inward cardiac sodium current, which will lead to prolonged QT interval. Three subtypes of ST-T segments exhibit distinct morphologies, and these features can be used to accurately predict genotype (24, 25).

### Literature screening

Traditional methods, editorials, and short reports for detecting LQTS were excluded. The final analysis was conducted by the same two authors (M-JW and W-QW) who reviewed all included studies. The chief investigator (X-WZ) resolved all disagreements between two authors about the selection of potential studies. Algorithms and ECG features from the studies that provided the most detailed information were retained. In order to compile and detect duplicates, EndNote X9.1 (Thomson Reuters) was used.

### Data extraction and quality assessment

We used a data extraction sheet to extract variables in our study. Literature searches and study selection were conducted prior to defining the data sheets. Publication year, author, target disease, task, data size, ECG features, algorithm type, and model performance metrics were extracted from each study.

Quality was assessed using the QUADAS-2 tool (26). To address the specifics of AI-related studies, QUADAS-AI tool was also used in our study (27). Each relevant article was independently assessed by two researchers (WZ and X-WZ) for bias risk and applicability concerns according to QUADAS-2 guidelines. Four domains (patient selection, index test, reference standard, flow and timing) of articles were assessed for bias using a range of questions. For each of the four domains, bias risk was estimated using a three-tailed scale: high, low, or unclear. A three-tailed scale was also used to assess applicability, with the exception of “flow and timing”. Consensus was used during scoring to resolve disagreements. All decisions were reached by consensus.

### Primary and subgroup analysis

We considered all studies in the full analysis dataset that tested ML models for detecting LQTS patients. A two-part analysis of the datasets was conducted: (a) training dataset; (b) test/validation dataset. LQTS types 1, 2, and 3 were also analyzed as subgroups (28).

### Statistical analysis

In this study, we analyzed the metrics (C-index and accuracy) for evaluating ML models. When the 95%CI and standard error (SE) of the C-index were missing, the SE was estimated according to Debray TP et al.'s research (29). ROC, sensitivity, specificity, PLR, NLR, DOR, and their 95%CI were computed (30, 31). Moderate accuracy is considered to be DOR > 25, and high accuracy is considered to be DOR > 100 (32, 33). For each test, sensitivity and specificity estimates were plotted on forest plots and in the ROC space as part of preliminary exploratory analyses. An AUC was determined, along with a 95% CI and a *P*-value. Heterogeneity was assessed using a forest plot, and significance was determined using Chi-squared and *I*^2^ statistics. Random effect models were used to estimate the effect size for all included studies, which helps reduce heterogeneity between them. Consequently, Bivariate mixed effects models were used to calculate pooled estimates. Subgroup analyses were conducted, as well as sensitivity analyses if necessary. In order to assess publication bias, logarithms of DOR were plotted against square roots of effective sample sizes; *P *< 0.05 for the slope coefficient indicates significant publication bias. Statistical analyses were conducted using Stata 15.0 (Stata Corp LP, College Station, TX) and the midas command.

## Results

### Study selection

**Figure 1:**
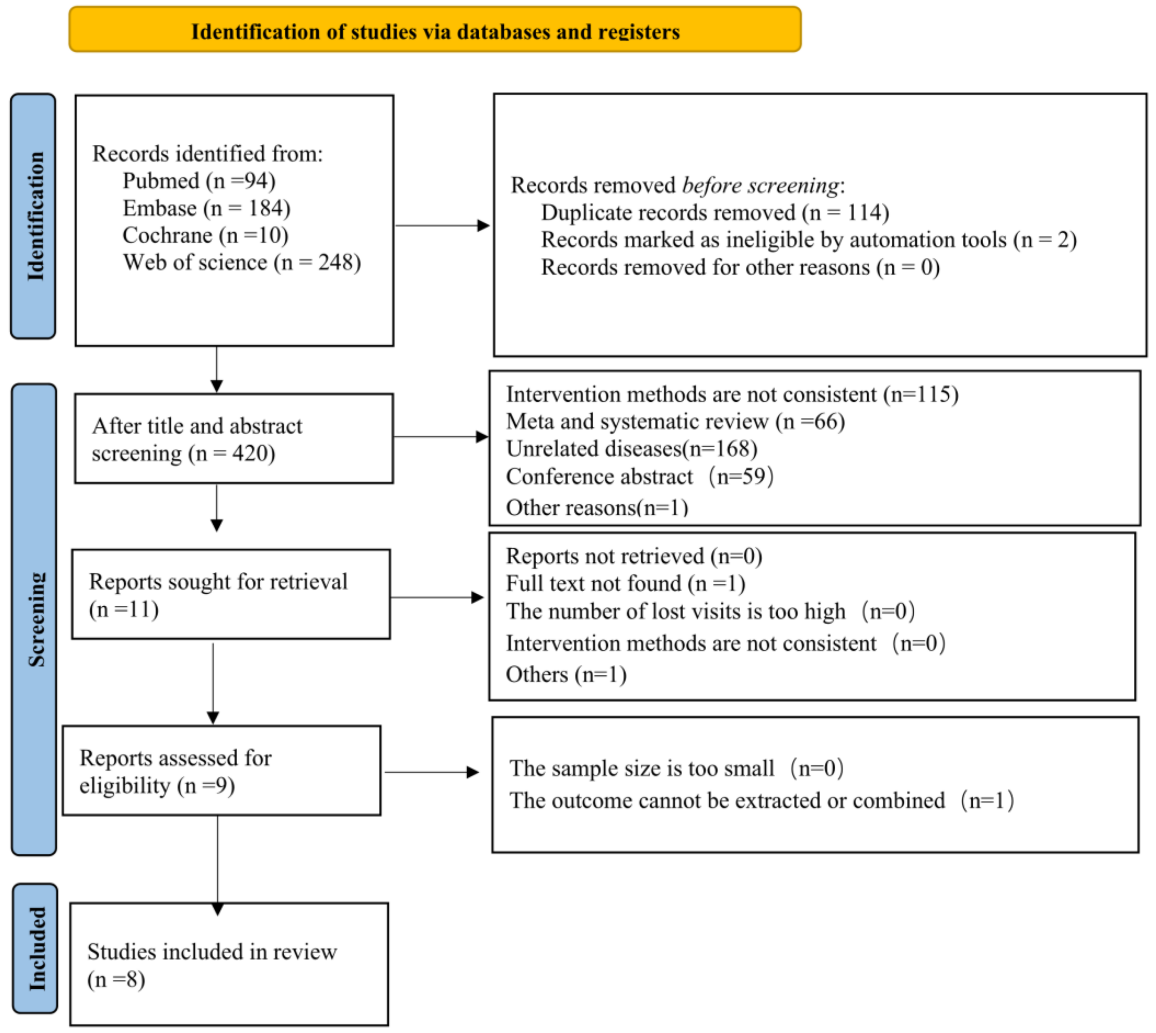
Flowchart of literature search.

In the initial search, 536 unique records were identified. In our study, 525 articles were excluded due to inclusion and exclusion criteria. The full-text review was performed on 11 studies, and 8 of these met all criteria for inclusion (Figure 1) (19, 21, 34–39).

### Study characteristics

Publication dates ranged from 2011 to 2022. There are eight studies to be reviewed across four regions: (1) Europe (*n* = 4), (2) North America (*n* = 2), (3) Oceania (*n* = 1), and (4) Asia (*n* = 1). The eight ML models included convolutional neural networks (CNNs) (*n* = 5), support vector machines (SVMs) (*n* = 2), and artificial neural networks (ANNs) (*n* = 1). In the majority of studies (*n* = 7) the ECG signals were used as inputs to the ML algorithm (19, 21, 34–37, 39), while in the remaining study (*n* = 1) the ECG signals were combined with demographic characteristics (age and gender) (38). Two studies analyzed data from multiple centers, while six studies examined data from a single center. Eight studies developed ML models in order to detect LQTS, and three of them differentiated genotypes further (19, 21, 34). The algorithm was evaluated using *k*-fold cross-validation in most studies (*n* = 6). The full analysis dataset included 16,584 participants. Cohort size ranged from 81 to 12,200. Study participants ranged in age from 21.60 ± 15.00 to 60.00 ± 18.00 years; 29.0% to 58.0% were males. LQTS patients were all over 16 years old. Finally, we included 2,692 LQTS patients and 14,417 controls: 42% were LQT1, 46% were LQT2, and 12% were LQT3. A total of 7,493,577 ECGs obtained from 12-lead ECGs were included in this study. The length of resting QTc ranged from 400 to 467 ms. There were 2,128, 362, and 202 LQTS patients in the training, test, and validation part, respectively (Table 1).

**Table 1:** Characteristics of included studies.

Study	Country	Dataset source	Population, *n*	Control group	Population in training, *n*	Population in test, *n*	Population in validation, *n*	Population with β-blocker, *n*
Aufiero (2022)	Netherlands	Amsterdam UMC University Hospital Leuven	LQTS: 570 Controls: 12,200	Genotype-negative patients with other possible cardiac disease	LQT1: 172 LQT2: 214 LQT3: 72	N/A	LQT1: 32 LQT2: 80 LQT3: 0	LQT1: 97 LQT2: 154 LQT3: 10
Bos (2021)	USA	Mayo Clinic	LQTS: 967 Controls: 1,092	Genotype-negative patients	LQTS: 587	LQT1: 149 LQT2: 109 LQT3: 32	LQTS: 90	N/A
Doldi (2021)	Germany	Single center	LQTS: 124 Controls: 161	Genotype-negative patients with other possible cardiac disease	LQT1: 65 LQT2: 44 LQT3: 12 LQT5: 3	N/A	N/A	N/A
Hajimolahoseini (2019)	Canada	Single center	LQTS: 45 Controls: 36	Genotype-negative patients	LQTS: 45	N/A	N/A	N/A
Hermans (2020)	Netherlands	Amsterdam UMC; University Hospital Leuven	LQTS: 333 Controls: 345	Genotype-negative family members	LQT1: 126 LQT2: 156 LQT3: 51	LQT1: 16 LQT2: 51 LQT3: 5	N/A	N/A
Hermans (2018)	Netherlands	MUSE Cardiology Information system	LQTS: 340 Controls: 348	Genotype-negative family members	LQT1: 129 LQT2: 160 LQT3: 51	N/A	N/A	N/A
Immanuel (2016)	Australia	THEW database	LQTS: 194 Controls: 140	Healthy individuals	LQT1: 133 LQT2: 61	N/A	N/A	LQT1: 36 LQT2: 23
Zeraatkar (2011)	Iran	MIT/BIH database	LQTS: 47 Controls: 95	Healthy individuals and TWA arrhythmia patients	LQTS: 47	N/A	N/A	N/A

Study	Dataset size	Number of leads	External validation	Algorithm evaluation	Prolonged QTc criteria	Feature	Length of resting QTc, ms
Aufiero (2022)	7, 481, 758 ECGs	12-lead ECGs	Y	5-fold cross-validation	≥450 ms for males ≥460 ms for females	*P* wave, QRS complex, S segment, T wave	LQT1: 456 ± 34 LQT2: 451 ± 36 LQT3: 450 ± 36
Bos (2021)	9,085 ECGs	12-lead ECGs	N	Holdout, 10% of dataset	≥450 ms	QT-Interval	LQTS: 467 ± 43
Doldi (2021)	730 ECGs	12-lead ECGs	N	5-fold cross-validation	≥450 ms for males ≥460 ms for females	RR-Interval, QT-Interval	LQTS: 465 ± 32
Hajimolahoseini (2019)	162 ECGs	12-lead ECGs	N	Cross-validation	>470 ms	QRS complex, R-R intervals	N/A
Hermans (2020)	678 ECGs	12-lead ECGs	Y	10-fold cross-validation	≥450 ms for males ≥460 ms for females	R peak, QRS complex, T-wave	Training: LQTS: 457 ± 38; LQT1: 455 ± 34; LQT2: 462 ± 36; LQT3: 446 ± 50 Test: LQTS: 456 ± 37; LQT1: 467 ± 44; LQT2: 455 ± 34; LQT3: 421 ± 11
Hermans (2018)	688 ECGs	12-lead ECGs	N	10-fold cross-validation	>480 ms	QT-interval, T-wave morphology, RR-interval	N/A
Immanuel (2016)	334 ECGs	12-lead ECGs	N	Holdout, 15% of dataset	≥450 ms	T-waves, R-wave amplitude, RR interval	400–450
Zeraatkar (2011)	142 ECGs	12-lead ECGs	N	Cross-validation	≥450 ms	QRS complex, T-wave	N/A

Study	Sensitivity	Specificity	AUROC	Algorithm
Aufiero (2022)	Training: LQT1: 0.840; LQT2: 0.900; LQT3: 0.970	Training: LQT1: 0.960; LQT2: 0.960; LQT3: 0.920	Training: LQT1: 0.900; LQT2: 0.920; LQT3: 0.890	CNN
	Validation: LQT1:0.870; LQT2: 0.900	Validation: LQT1: 0.930; LQT2: 0.880	Validation: LQT1: 0.900; LQT2: 0.890	
Bos (2021)	Training: LQTS: 0.837	Training: LQTS: 0.806	Training: LQTS: 0.900	CNN
	Test: LQTS: 0.837; LQT1: 0.846; LQT2: 0.881; LQT3: 0.781	Test: LQTS: 0.854; LQT1: 0.872; LQT2: 0.895; LQT3: 0.802	Test: LQTS: 0.914; LQT1: 0.921; LQT2: 0.944; LQT3: 0.863	
	Validation: LQTS: 0.790	Validation: LQTS: 0.787	Validation: LQTS: 0.862	
Doldi (2021)	Training: LQTS: 0.908	Training: LQTS: 0.929	Training: LQTS: 0.970	CNN
Hajimolahoseini (2019)	Training: LQTS: 0.960	Training: LQTS: 0.970	N/A	CNN
Hermans (2020)	Training: LQTS: 0.830	Training: LQTS: 0.850	Training: LQTS: 0.990	SVM
	Test: LQTS: 0.750	Test: LQTS: 0.840	Test: LQTS: 0.840	
Hermans (2018)	Training: LQTS: 0.820	Training: LQTS: 0.861	Training: LQTS: 0.901	SVM
Immanuel (2016)	Training: LQTS: 0.943; LQT1: 0.882	Training: LQTS: 0.822; LQT1: 0.889	Training: LQTS: 0.880; LQT1: 0.710	CNN
Zeraatkar (2011)	Training: LQTS: 0.997	Training: LQTS: 0.998	N/A	ANN

CNN, convolutional neural network; ANN, artificial neural network; SVM, support vector machine; Y, yes; N, no.

### Risk of bias assessment

Detailed quality assessment results are presented in Supplementary Table S2. Providing that the source, size, and input data quality were accurately characterized, along with clear criteria for patient eligibility, a low risk of bias was observed in the “patient selection” domain. The index test was judged primarily on the validity of models on external datasets. Diagnostic performance may be overestimated if the model is tested exclusively on internal data, the “index test” domain lacked external validation, resulting in a high risk of bias. Additionally, the absence of external validation adversely affected the level of concern regarding applicability, as the results of diagnostic effectiveness cannot be extrapolated to the general population based on an in-house data set alone. The same reference standard was used in all studies. The review question matched the target conditions defined by a reference standard. All included studies described genetic testing as the reference standard. The timing between the index test and the reference standard was often inaccurate. Most studies, however, provided positive information concerning these indicators.

### Machine learning model and LQTS detection

**Figure 2:**
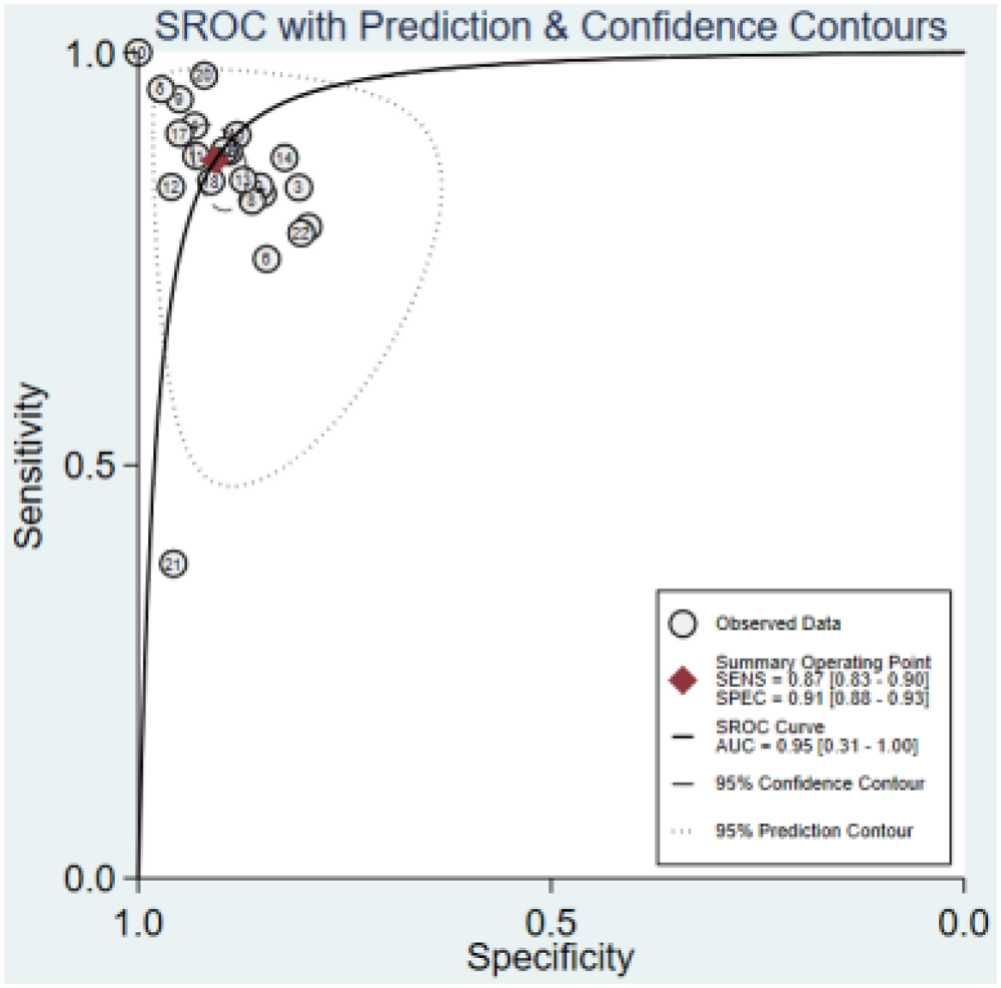
SAUROC of ML algorithms for detecting LQTS.

In eight studies, the performance of ML approaches was examined for detecting LQTS patients. ML was found to be an effective method for diagnosing LQTS in this meta-analysis. According to the full analysis dataset, the ML models performed well in identifying LQTS, and the overall pooled AUROC for ML to identify LQTS was 0.95 (95% CI: 0.31–1.00) (Figure 2).

**Figure 3:**
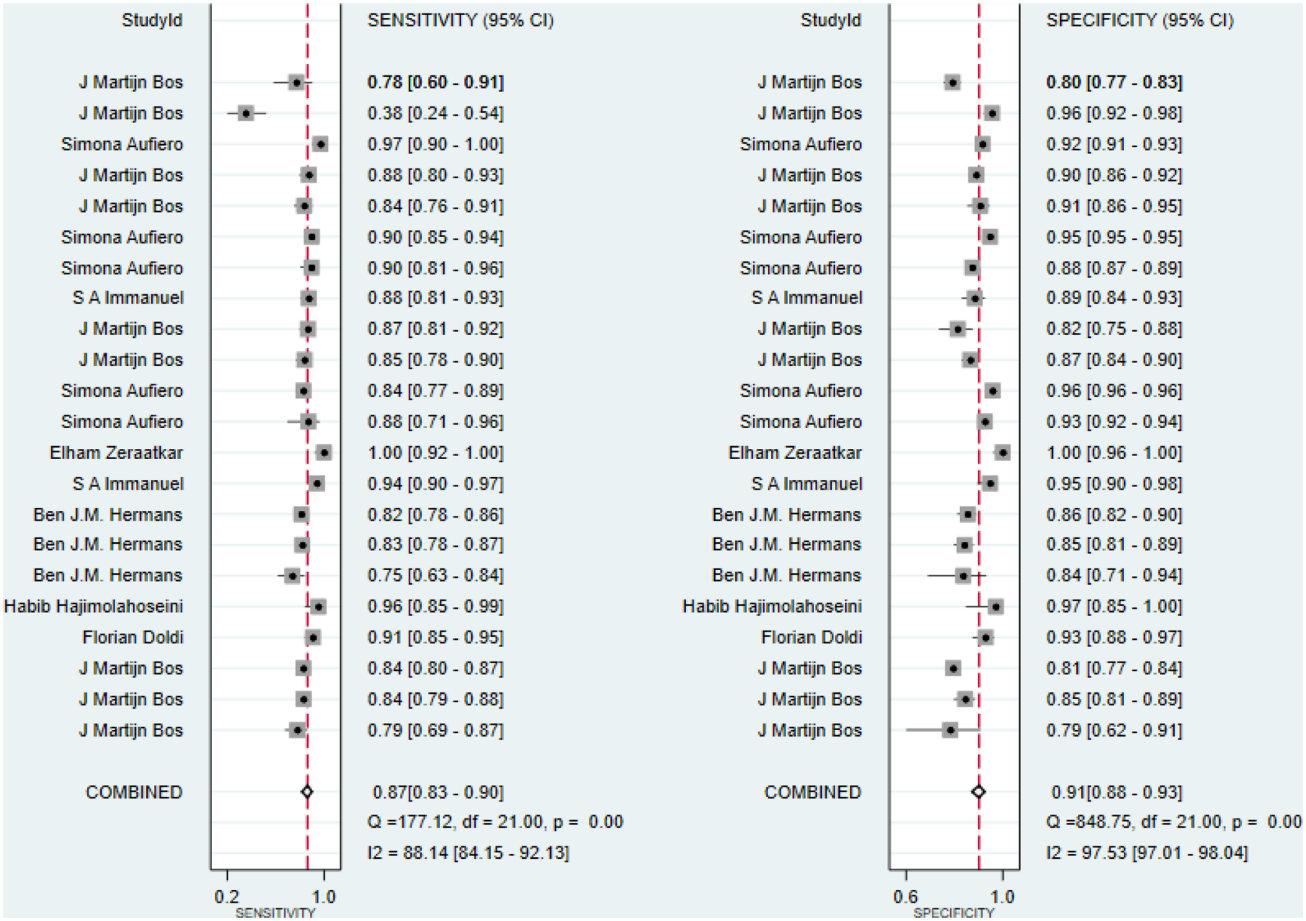
Sensitivity and specificity of ML algorithms for detecting LQTS.

**Figure 4:**
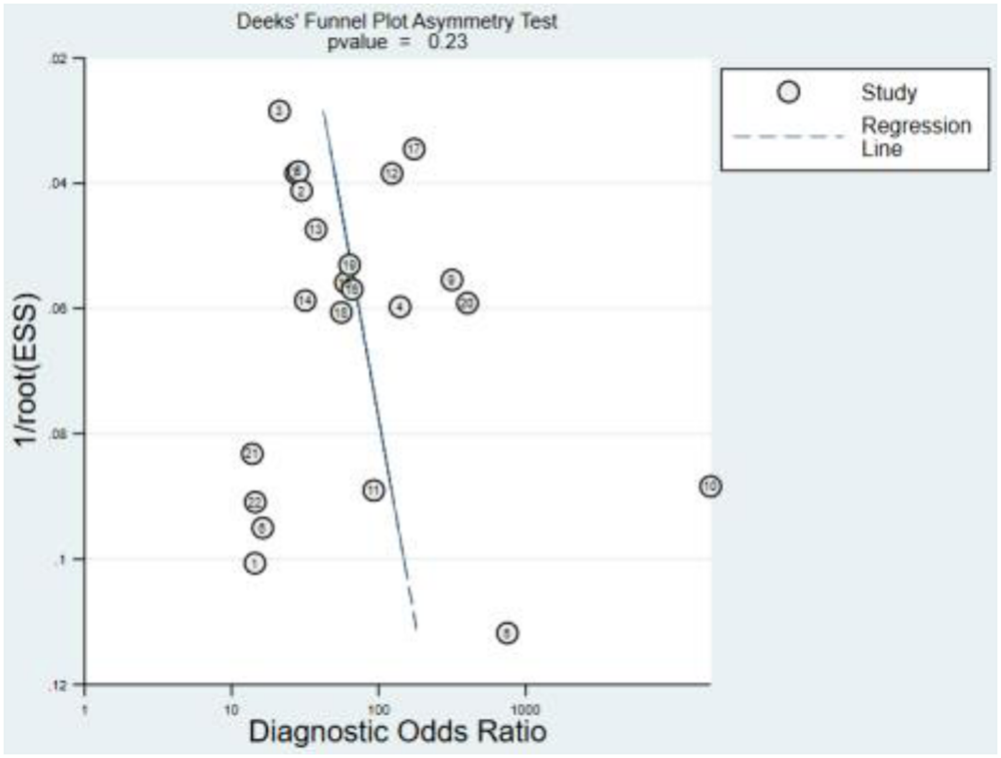
DOR of ML algorithms for detecting LQTS.

Additionally, sensitivity, specificity, and DOR were 0.87 (95% CI: 0.83–0.90), 0.91 (95% CI: 0.88–0.93), and 65 (95% CI: 39–109), respectively (Figures 3, 4). Fagan nomogram showed that a positive test increases significantly the pretest probability of LQTS, from 20% to 70%, while a negative test decreases significantly the pretest probability, from 20% to 3% (Supplementary Figure S3). PLR was 9.3 (95% CI: 7.0–12.3) and NLR was 0.14 (95% CI: 0.11–0.20), with very low heterogeneity (*I*^2 ^= 16%) (Supplementary Figures S4, S5). Asymmetry was not found in Deeks' funnel plots for publication bias. The publication bias coefficient was 17.60, which was not significant (*P* = 0.23). All studies did not demonstrate publication bias.

### Training dataset

The overall pooled SAUROC for ML to detect LQTS was 0.96 (95% CI: 0.32–1.00) (Supplementary Figure S6). Sensitivity, specificity, and DOR were 0.90 (95% CI: 0.86–0.93), 0.93 (95% CI: 0.89–0.96), and 125 (95% CI: 55–285), respectively (Supplementary Figures S7, S8). Fagan nomogram showed that a positive test increases significantly the pretest probability of LQTS, from 20% to 77%, while a negative test decreases significantly the pretest probability, from 20% to 3% (Supplementary Figure S9). The overall pooled PLR was 13.4 (95% CI: 8.0–22.4) and the NLR was 0.11 (95% CI: 0.07–0.16), with medium heterogeneity (*I*^2 ^= 62%) (Supplementary Figures S10, S11). There was no asymmetry in Deeks' funnel plot for publication bias as well. However, the publication bias evaluation indicated that the bias coefficient was 59.68 and significant (*P* = 0.01).

### Test and validation dataset

The ability of ML to detect LQTS in the test/validation dataset was evaluated in three studies. The overall pooled SAUROC for diagnosing LQTS was 0.92 (95% CI: 0.29–1.00) (Supplementary Figure S12). The sensitivity, specificity, and DOR were 0.82 (95% CI: 0.75–0.87), 0.89 (95% CI: 0.85–0.91), and 34 (95% CI: 24–49), respectively (Supplementary Figures S13, S14). Fagan nomogram showed that a positive test increases significantly the pretest probability of LQTS, from 20% to 64%, while a negative test decreases significantly the pretest probability, from 20% to 5% (Supplementary Figure S15). The PLR was 7.1 (95% CI: 5.6–9.0), and the NLR was 0.21 (95% CI: 0.15–0.28), with low heterogeneity (*I*^2 ^= 37%) (Supplementary Figures S16, S17). Publication bias was evaluated, and a bias coefficient of −11.04 was found to be non-significant (*P* = 0.26). Test/validation datasets did not demonstrate publication bias.

### Subgroup analysis

In order to evaluate ML detection performance for LQTS subtypes, subgroup analysis was conducted (Table 2). LQT3 was not studied extensively, so we only examined ML's performance in identifying LQT1 and LQT2.

**Table 2:** Performance of detecting LQTS subtypes.

Type	AUROC	Sensitivity (95%CI)	Specificity (95%CI)	PLR (95%CI)	NLR (95%CI)	DOR (95%CI)
LQT1	0.89	0.86 (0.83–0.88)	0.91 (0.86–0.94)	9.4 (6.0–14.8)	0.16 (0.13–0.19)	60 (37–99)
LQT2	0.92	0.88 (0.85–0.91)	0.91 (0.88–0.94)	10.2 (7.1–14.8)	0.13 (0.10–0.17)	79 (45–138)

#### LQT1

Three studies assessed ML's performance for identifying LQT1. The overall pooled AUROC for identifying LQT1 was 0.89 (95% CI: 0.16–1.00). Additionally, the sensitivity, specificity, DOR were 0.86 (95%CI: 0.83–0.88), 0.91 (95%CI: 0.86–0.94), and 60 (95% CI: 37–99). The PLR was 9.4 (95% CI: 6.0–14.8), and the NLR was 0.16 (95% CI: 0.13–0.19).

#### LQT2

Two studies assessed ML's performance for identifying LQT2. The overall pooled AUROC for identifying LQT2 was 0.92 (95% CI: 0.18–1.00). Additionally, the sensitivity, specificity, DOR were 0.88 (95%CI: 0.85–0.91), 0.91 (95%CI: 0.88–0.94), and 79 (95% CI: 45–138). The PLR was 10.2 (95% CI: 7.1–14.8), and the NLR was 0.13 (95% CI: 0.10–0.17).

## Discussion

ML algorithms for the detection of LQTS were investigated through this meta-analysis. The overall pooled estimation indicated that ML performed well in recognizing LQTS; thus, we believe that pooling the diagnostic accuracy from eligible studies would be beneficial to cardiologists and researchers.

### Performance and clinical relevance of models

Providing appropriate treatment for LQTS requires accurate identification (6). As of today, LQTS has been diagnosed not by genetic testing, but by the Schwartz Score, along with ECGs and clinical parameters that can be used in conjunction with genetic testing (2). Genetic testing is recommended if clinical presentation, family history, and ECG characteristics indicate LQTS, but its high cost and time delay limit its application. Exercise and QT-stand testing may lead to overdiagnosis of LQTS in children and adolescents with low likelihoods of the illness (40). For the diagnosis of LQTS, the 12-lead ECGs is still the most essential diagnostic tool. Studies have shown that the present ECG-based ML models play a significant role in diagnosis and genotyping of LQTS with excellent accuracy. This could mean that ML models could be implemented in clinical care, for example, it could potentially serve as a clinical decision tool to help general cardiologists predict which patients might need further workup.

### Pooled performance and heterogeneity

LQTS patients were identified by genetic testing in all the included studies. To estimate the overall performance of ML, AUROC values were pooled across all models presented. LQTS genotypes were identified and distinguished using ML algorithms with high sensitivity and specificity; the PLR, NLR, and DOR values indicate effective test performance. All accuracy values were above 0.89, which suggested that ECG-based ML models were effective in predicting LQTS. However, the CI was very broad and the AUC estimate was skewed to the upper end of the 95% CI, possibly due to a lack of available studies. One of the most common reasons for a broadening CI is an insufficient sample size. Our study only included a total of 2,692 LQTS patients, which may make the CI too broad. Another explanation may be the wide variation in the standard errors of the included models (ranging from 0.008 to 0.034). Besides, AUC values of training datasets are generally higher than those of test/validation datasets. Only two included studies were tested on the test/validation dataset, so the overall pooled AUROC of our study mainly comes from the training dataset, which may explain the higher AUC estimate.

This meta-analysis was conducted using a subgroup analysis to resolve heterogeneity between studies. However, model performance differed significantly between studies. It was found that the models tested on the full analysis dataset (AUROC of 0.95) performed less well than those tested on the training dataset (AUROC of 0.96), while they performed better than those tested on the test/validation dataset (AUROC of 0.92). Based on the fact that two studies of the test/validation dataset used external validation data, we assumed that the sources of heterogeneity were derived from different population groups.

### Performance of subgroup analysis

In the eight included studies, the pooled estimation was better in the training dataset compared to the test/validation dataset after dividing them into two categories according to the different datasets, indicating ML models require more external verification in order to prevent overestimation caused by overfitting and bias (41). Only Aufero and Hermans' model was externally validated among all the included studies (34, 37). Validation datasets are used to tune hyperparameters, while test datasets are used to evaluate the final model fit unbiasedly (42). It is more common for ML models to be validated internally by using test datasets derived from the training dataset rather than externally by using independent datasets.

Also, we analyzed the performance of ML models for identifying LQTS subtypes. ML models are more accurate in detecting LQT2 than LQT1. Bos et al. used unsupervised feature extraction, in which all 12-lead ECG waveform were analyzed agnostically during training. The LQTS subtypes of this model were subsequently classified using this unsupervised training model. Considering the nature of CNNs, they could not identify which ECG features were critical for identifying LQTS subtypes (21). Aufiero et al. assumed that QRS features are the main ECG signal for classification as their ML models derive much from the initial part of the QRS complex (34).

### Clinical model performance

Even though genetic testing is an effective method for identifying genetic heterogeneity, it cannot definitively exclude LQTS as a diagnosis (43). 12-lead ECGs is recommended as a basis for diagnosing LQTS (28, 44). Therefore, it is imperative to investigate whether ECG-based ML models could improve patient outcomes. We distinguished between studies comparing the performance of ML models with that of expert cardiologists. There were only two studies that validated their models and showed that they were almost as accurate as experts at providing diagnostic information (34, 37). ML models were in good agreement with experts with regard to sensitivity and specificity. Theoretically, ML models could not replace experts, but could be used to assist physicians with limited knowledge of LQTS (37).

### Future directions and academic contribution

The fact that many models were developed using similar populations should be noted. Four models were developed based on patients predominantly of European ancestry, with three models developed in the Netherlands and only one model developed in Iran. It is known that LQTS genes are influenced by ancestry in terms of diversity and prevalence of genetic variants (45). Prior studies have demonstrated that arrhythmia-associated sequence variants as well as polymorphisms vary significantly by ethnicity (46–48). According to a study that assessed KCNQ1 (LQT1) and KCNH2 (LQT2) variants in 744 individuals, 86% (42 of 49) of them were ethnicity-specific and were found exclusively in Asians (*n* = 2), Hispanics (*n* = 2), African Americans (*n* = 26), and Europeans (*n* = 12) (46). European ancestry showed higher frequencies of LQT1 and 2 compared to other ethnicities. It is most prevalent among African Americans (4.5%) who carry the rare variant of the SCN5A (LQT3) gene (47). SCN5A variants were found in 829 individuals, with 49% of them being African Americans. In particular, among African Americans, 13% have a variant of SCN5A related to increased arrhythmia risks, while neither Asians nor Europeans do (46). To ensure generalization, we encourage the development of models based on data from different centers and countries.

Further research is needed to determine the most effective strategies for integrating ML models into clinical workflows and to assess the effectiveness of these strategies on relevant clinical outcomes. Several factors, including sex, age, and comorbidities, may contribute to clinically relevant heterogeneity in the detection of LQTS (24). With the use of ML models, it can be possible to investigate whether or not it is possible to identify the precise population using pre-specified parameters in order to administer treatment more effectively to groups of patients that are more homogeneous.

### Strengths and limitations

A number of strengths can be found in our study. In this study, we attempted to investigate the potential of ML models in the diagnosis and genotyping of LQTS. A comprehensive search strategy and an in-depth analysis were employed. Any model used to identify LQTS was included, which permitted us to include models that were not originally intended for detecting LQTS but may have merit. ML models for the two most common LQTS subtypes were also analyzed by subgroup meta-analysis, enhancing the relevance of our study.

According to the studies we included, we were limited by the data they reported. Our meta-analysis has the following limitations: (1) LQTS patients were identified using various types of ML models in all the included studies. As a result, we are unable to suggest which model was most suitable for detecting LQTS based on the results of our study. Nevertheless, all of the models examined in the included studies demonstrated that ML algorithms can assist cardiologists in detecting LQTS. (2) Only two studies were externally validated, which inherently limited the quality of evidence. The outcome of this study would have been improved if all studies had been externally validated. (3) As LQTS is relatively rare, we enrolled only 2,692 patients with LQTS in our analysis, mostly with LQT1 or LQT2, and only 12% with LQT3, so further evaluation is necessary to determine the performance of ML models for LQTS subtypes. (4) As β-blocker will shorten the QT interval, ML algorithms performance may differ on ECGs where individuals are treated with β-blocker. In our study, only Aufero and Hermans reported the number of LQTS patients with β-blocker therapy (19, 34). However, like another six studies, they also did not separate groups based on β-blocker treatment due to the lack of sample size. Thus, it will be important to investigate whether the discriminatory power of ML algorithms can be replicated in an independent cohort of LQTS patients. (5) For diagnosing LQTS, T-wave morphology is most commonly used. Most researchers in our study also implemented ML algorithms to identify the differentiation of LQTS and controls based on T-wave morphology features after calculating the length of QTc (19, 21, 34, 35, 37–39). They extracted features from T-wave, such as the Q to T-peak, the T-peak to T-end interval, T-wave magnitude, and T-loop slopes to differentiate LQTS from other cardiovascular diseases with similar T-wave. However, only 3 included studies explicitly reported that the patients in control group had other possible cardiac disease, restricting analysis of ML algorithms’ diagnostic value in individuals with any underlying cardiovascular disease.

## Conclusion

ML algorithms for the diagnosis of LQTS are currently being developed, but they are gradually evolving into a form that can be applied to clinical practice. Clinically, however, the development of algorithms for identifying LQTS subtypes is critical. In addition, external validation of ML algorithms in LQTS is crucial. ML algorithms could be popularized and applied to clinical practice by cardiologists once they are validated in this manner.

## Data Availability

The raw data supporting the conclusions of this article will be made available by the authors, without undue reservation.
