# Supplementary material for: The diagnostic value of electrocardiogram-based machine learning in long QT syndrome: a systematic review and meta-analysis
Source: Front Cardiovasc Med. 2023 Jun 7;10:1172451. doi: 10.3389/fcvm.2023.1172451 (PMC10282180; doi:10.3389/fcvm.2023.1172451)

**Supplementary Appendix**

**The diagnostic value of electrocardiogram-based machine learning in long QT syndrome: a systematic review and meta-analysis**

**Minjuan Wu et al.**

**Table of Contents**

**Supplementary Table S1. Search strategy in PubMed, Cochrane, Embase, and the Web of Science from database inception to inception to August 16^th^, 2022**

**Supplementary Table S2. Quality Assessment of Diagnostic Accuracy Studies–AI (QUADAS-AI)**

**Supplementary figure S3. Fagan nomogram used to illustrate the posttest probabilities of LQTS**

**Supplementary figure S4. PLR and NLR of machine learning models for detecting LQTS**

**Supplementary figure S5. Heterogeneity of machine learning models for detecting LQTS**

**Supplementary figure S6. SAUROC of machine learning models for detecting LQTS (training dataset)**

**Supplementary figure S7. Sensitivity and specificity of machine learning models for detecting LQTS (training dataset)**

**Supplementary figure S8. DOR of machine learning models for detecting LQTS (training dataset)**

**Supplementary figure S9. Fagan nomogram used to illustrate the posttest probabilities of LQTS (training dataset)**

**Supplementary figure S10. PLR and NLR of machine learning models for detecting LQTS (training dataset)**

**Supplementary figure S11. Heterogeneity of machine learning models for detecting LQTS (training dataset)**

**Supplementary figure S12. SAUROC of machine learning models for detecting LQTS (test/validation dataset)**

**Supplementary figure S13. Sensitivity and specificity of machine learning models for detecting LQTS (test/validation dataset)**

**Supplementary figure S14. DOR of machine learning models for detecting LQTS (test/validation dataset)**

**Supplementary figure S15. Fagan nomogram used to illustrate the posttest probabilities of LQTS (test/validation dataset)**

**Supplementary figure S16. PLR and NLR of machine learning models for detecting LQTS (test/validation dataset)**

**Supplementary figure S17. Heterogeneity of machine learning models for detecting LQTS (test/validation dataset)**

**Table S1. Search strategy in PubMed, Cochrane, Embase, and the Web of Science from database inception to inception to August 16^th^, 2022**

#1 Long QT Syndrome [Mesh]

#2 Long QT Syndrome [Title/Abstract]

#3 Electrocardiogram QT Prolonged [Title/Abstract]

#4 long Q-T syndrome [Title/Abstract]

#5 LQTS [Title/Abstract]

#6 OR/1-5

#7 Jervell-Lange Nielsen Syndrome [Mesh]

#8 Jervell-Lange Nielsen Syndrome [Title/Abstract]

#9 Jervell and Lange-Nielsen syndrome [Title/Abstract]

#10 Jervell Lange Nielsen Syndrome [Title/Abstract]

#11 Syndrome, Jervell-Lange Nielsen [Title/Abstract]

#12 Cardioauditory Syndrome of Jervell and Lange-Nielsen [Title/Abstract]

#13 Cardioauditory Syndrome of Jervell and Lange Nielsen [Title/Abstract]

#14 Cardio-Auditory-Syncope Syndrome [Title/Abstract]

#15 Cardio Auditory Syncope Syndrome [Title/Abstract]

#16 Cardio-Auditory-Syncope Syndromes [Title/Abstract]

#17 Syndrome, Cardio-Auditory-Syncope [Title/Abstract]

#18 Syndromes, Cardio-Auditory-Syncope [Title/Abstract]

#19 Deafness, Congenital, and Functional Heart Disease [Title/Abstract]

#20 Prolonged QT Interval in EKG and Sudden Death [Title/Abstract]

#21 Jervell and Lange-Nielsen Syndrome [Title/Abstract]

#22 Jervell and Lange Nielsen Syndrome [Title/Abstract]

#23 Surdo-Cardiac Syndrome [Title/Abstract]

#24 Surdo Cardiac Syndrome [Title/Abstract]

#25 Surdo-Cardiac Syndromes [Title/Abstract]

#26 Syndrome, Surdo-Cardiac [Title/Abstract]

#27 Jervell And Lange-Nielsen Syndrome 1 [Title/Abstract]

#28 Jervell And Lange Nielsen Syndrome 1 [Title/Abstract]

#29 congenital deafness with syncope and prolonged qt interval [Title/Abstract]

#30 Jervell Lange Nielsen syndrome [Title/Abstract]

#31 Jervell Lange Nielson syndrome [Title/Abstract]

#32 Jervell Nielsen syndrome [Title/Abstract]

#33 Jervell-Lange Nielsen syndrome [Title/Abstract]

#34 Lange Nielson Jervell syndrome [Title/Abstract]

#35 prolonged qt interval with syncope and congenital deafness [Title/Abstract]

#36 OR/7-35

#37 Romano-Ward Syndrome [Mesh]

#38 Romano-Ward Syndrome [Title/Abstract]

#39 Romano Ward Syndrome [Title/Abstract]

#40 Syndrome, Romano-Ward [Title/Abstract]

#41 Ventricular Fibrillation with Prolonged QT Interval [Title/Abstract]

#42 Long QT Syndrome Type 1 [Title/Abstract]

#43 Long QT Syndrome 1 [Title/Abstract]

#44 Ward-Romano Syndrome [Title/Abstract]

#45 Syndrome, Ward-Romano [Title/Abstract]

#46 Ward Romano Syndrome [Title/Abstract]

#47 Romano Ward ECG [Title/Abstract]

#48 OR/37-47

#49 Andersen Syndrome [Mesh]

#50 Andersen Syndrome [Title/Abstract]

#51 Syndrome, Andersen [Title/Abstract]

#52 Andersen Cardiodysrhythmic Periodic Paralysis [Title/Abstract]

#53 Potassium-Sensitive Periodic Paralysis, Ventricular Ectopy, and Dysmorphic Features [Title/Abstract]

#54 Andersen Tawil Syndrome [Title/Abstract]

#55 Syndrome, Andersen Tawil [Title/Abstract]

#56 Long QT Syndrome 7 [Title/Abstract]

#57 Periodic Paralysis, Potassium-Sensitive Cardiodysrhythmic Type [Title/Abstract]

#58 Periodic Paralysis, Potassium Sensitive Cardiodysrhythmic Type [Title/Abstract]

#59 Andersen-Tawil Syndrome [Title/Abstract]

#60 Andersen Cardiodysrythmic Periodic Paralysis [Title/Abstract]

#61 Andersen Tawil syndrome [Title/Abstract]

#62 Andersen triad [Title/Abstract]

#63 Andersen's syndrome [Title/Abstract]

#64 Andersens syndrome [Title/Abstract]

#65 long QT syndrome 7 [Title/Abstract]

#66 LQT 7 [Title/Abstract]

#67 OR/49-66

#68 machine learning [Mesh]

#69 machine learning [Title/Abstract]

#70 Transfer Learning [Title/Abstract]

#71 Deep learning [Title/Abstract]

#72 Learning, Transfer [Title/Abstract]

#73 Ensemble Learning [Title/Abstract]

#74 artificial intelligence [Title/Abstract]

#75 Prediction model [Title/Abstract]

#76 random forest [Title/Abstract]

#77 neural network [Title/Abstract]

#78 Support vector machine [Title/Abstract]

#79 SVM [Title/Abstract]

#80 Gradient Boosting Machine [Title/Abstract]

#81 GBM [Title/Abstract]

#82 Nomogram [Title/Abstract]

#83 XGboost [Title/Abstract]

#84 Adaboost [Title/Abstract]

#85 Decision tree [Title/Abstract]

#86 Development and validation [Title/Abstract]

#87 OR/68-86

#88 #6 AND #36 AND #48 AND #67 AND #87

**Table S2. Quality Assessment of Diagnostic Accuracy Studies-AI (QUADAS-AI)**

|  | **Risk of Bias** | | | | **Applicability Concerns** | | |
| --- | --- | --- | --- | --- | --- | --- | --- |
| **Study** | **Patient selection** | **Index**  **test** | **Reference standard** | **Flow and timing** | **Patient selection** | **Index test** | **Reference standard** |
| Aufiero (2022) | L | L | L | L | L | L | L |
| Bos (2021) | L | H | L | L | L | H | L |
| Doldi (2021) | L | H | L | L | L | H | L |
| Hajimolahoseini (2019) | L | H | L | L | L | H | L |
| Hermans (2020) | L | L | L | L | L | L | L |
| Hermans (2018) | L | H | L | L | L | H | L |
| Immanuel (2016) | L | H | L | L | L | H | L |
| Zeraatkar (2011) | L | H | L | U | L | H | L |

H: high risk of bias; L: low risk of bias; U: Unclear risk of bias.

**Supplementary figure S3.** **Fagan nomogram used to illustrate the posttest probabilities of LQTS**


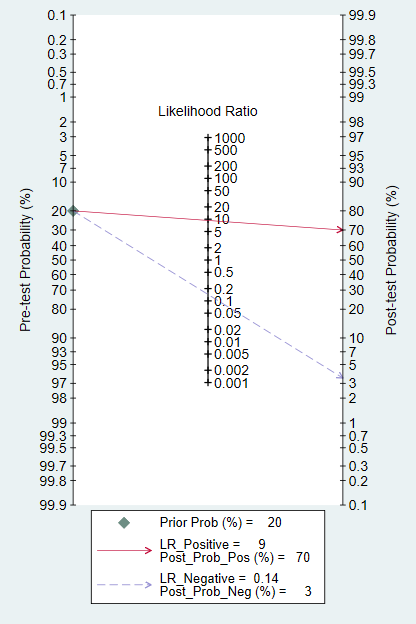


**Supplementary figure S4. PLR and NLR of machine learning models for detecting LQTS**


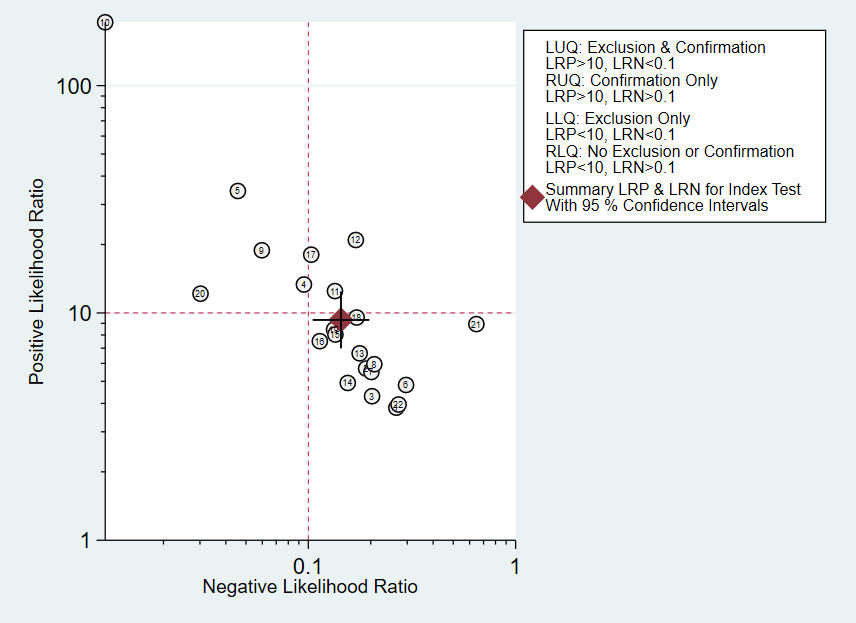


**Supplementary figure S5. Heterogeneity of machine learning models for detecting LQTS**


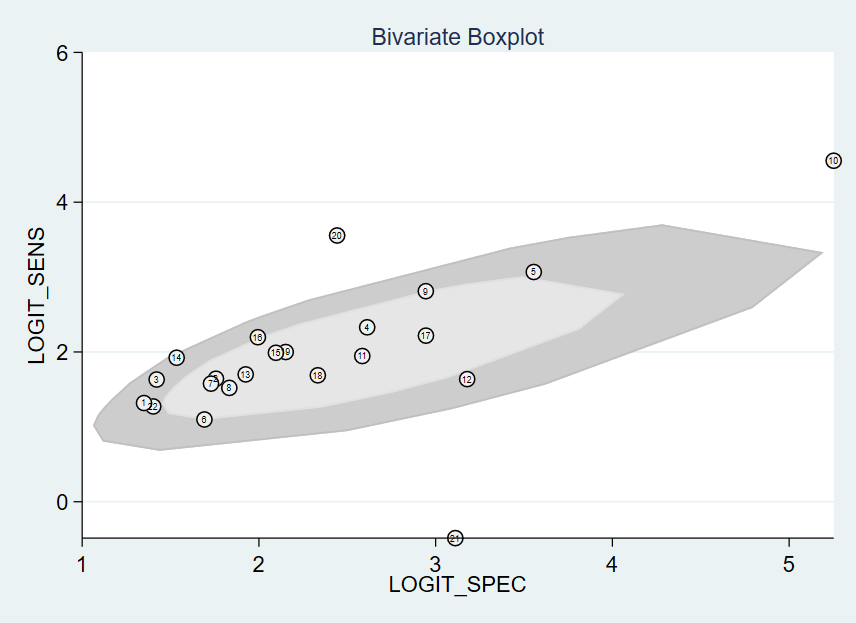


**Supplementary figure S6. SAUROC** **of machine learning models for detecting LQTS (training dataset)**


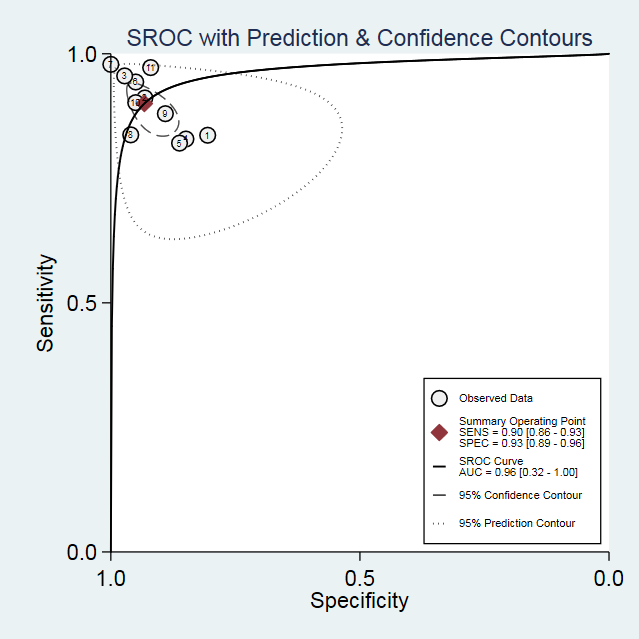


**Supplementary figure S7. Sensitivity and specificity of machine learning models for detecting LQTS (training dataset)**


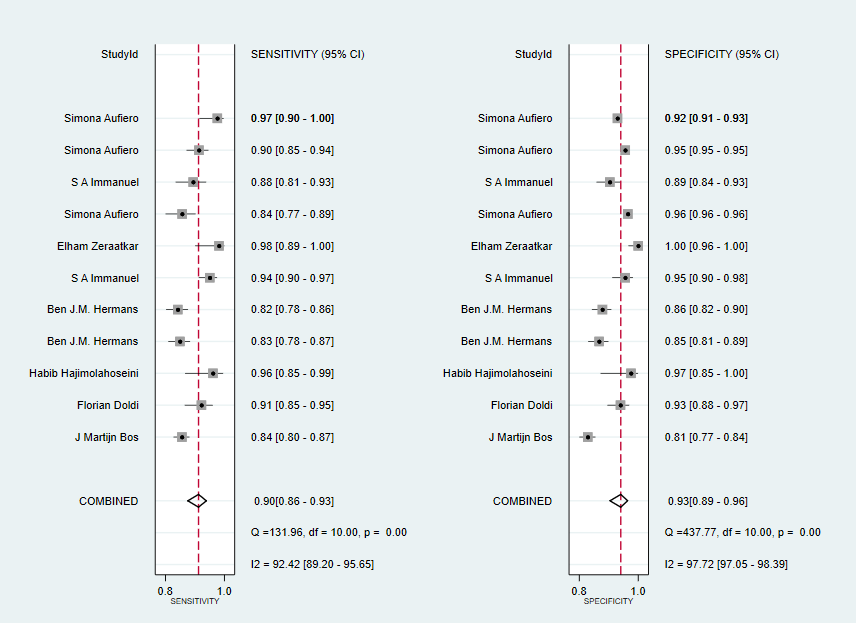


**Supplementary figure S8. DOR of machine learning models for detecting LQTS (training dataset)**


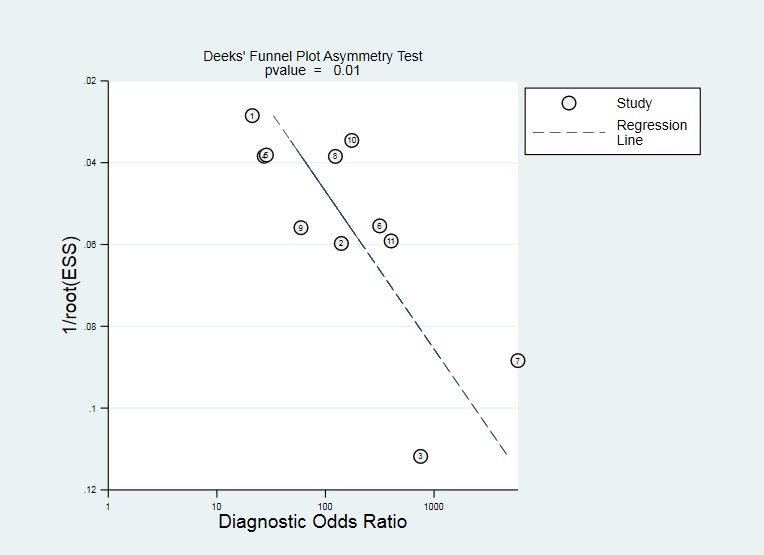


**Supplementary figure S9. Fagan nomogram used to illustrate the posttest probabilities of LQTS (training dataset)**


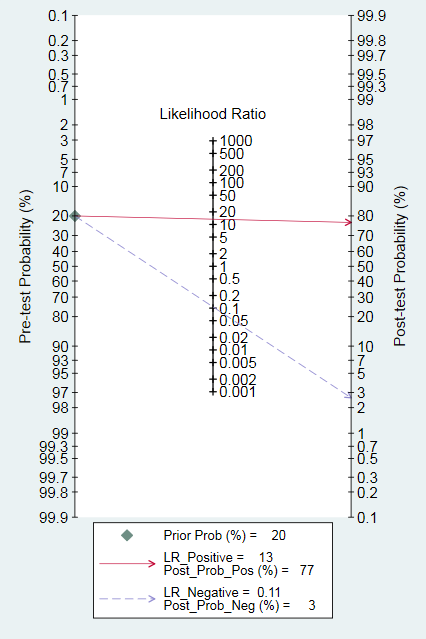


**Supplementary figure S10. PLR and NLR of machine learning models for detecting LQTS (training dataset)**


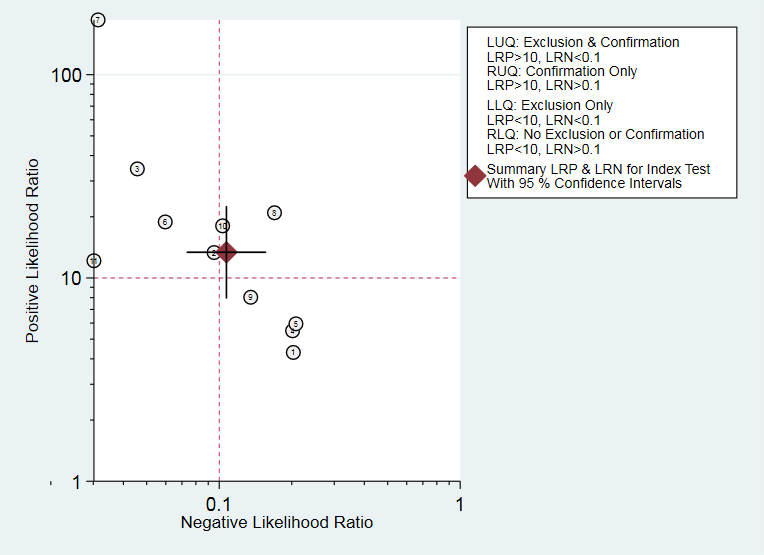


**Supplementary figure S11. Heterogeneity of machine learning models for detecting LQTS (training dataset)**


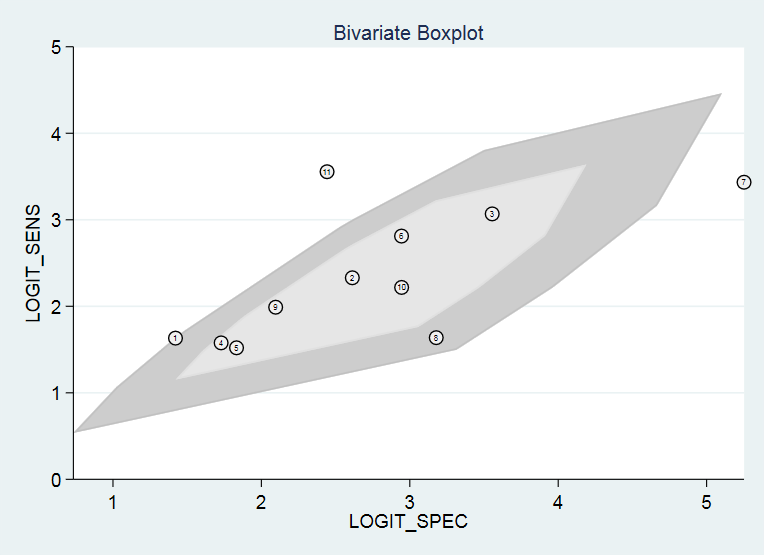


**Supplementary figure S12. SAUROC of machine learning models for detecting LQTS (test/validation dataset)**


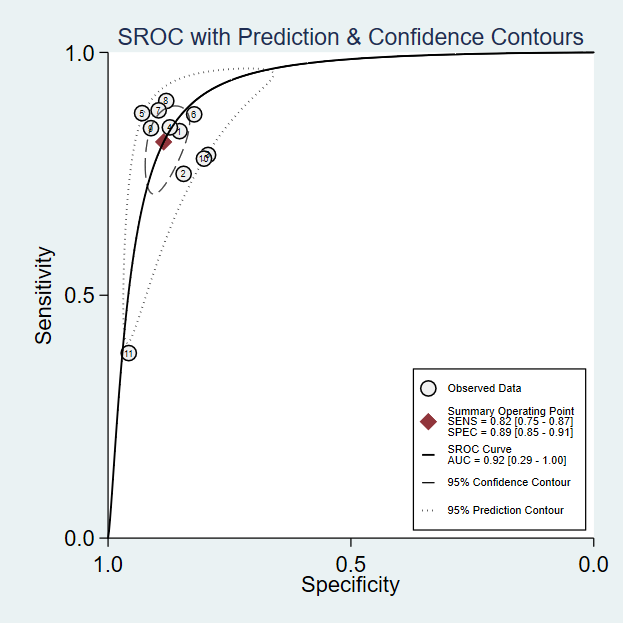


**Supplementary figure S13. Sensitivity and specificity of machine learning models for detecting LQTS (test/validation dataset)**


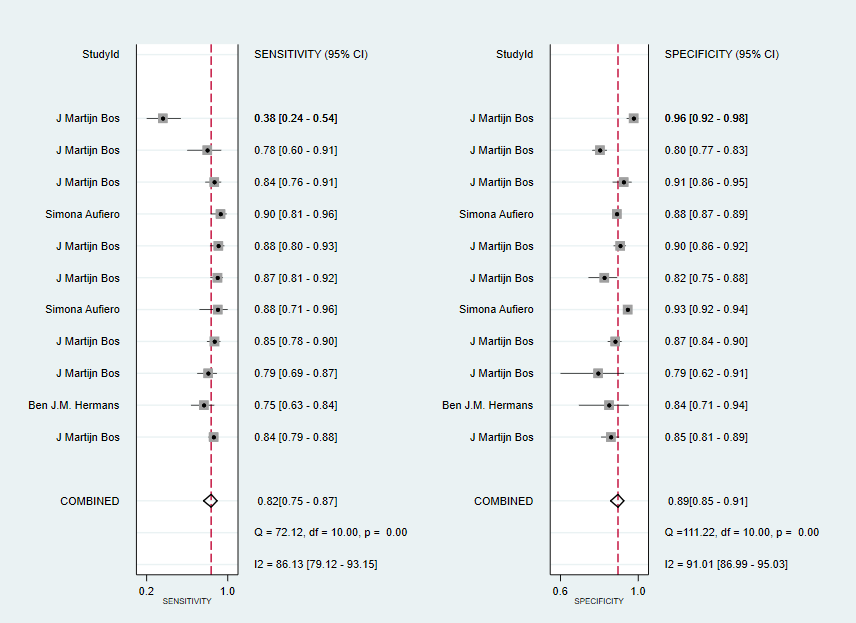


**Supplementary figure S14. DOR of machine learning models for detecting LQTS (test/validation dataset)**


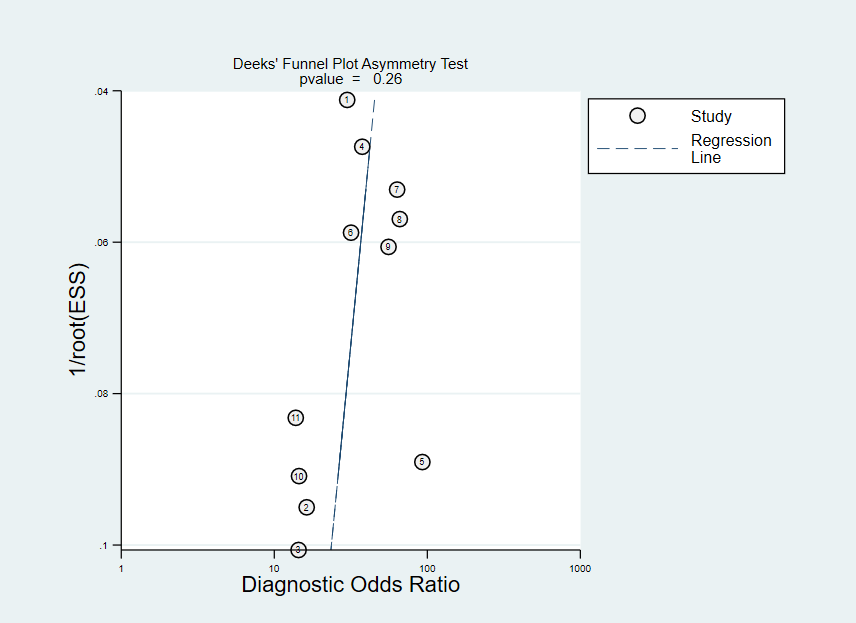


**Supplementary figure S15.** **Fagan nomogram used to illustrate the posttest probabilities of LQTS** **(test/validation dataset)**


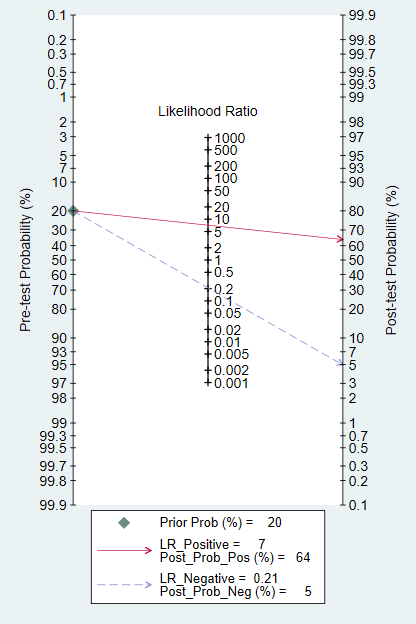


**Supplementary figure S16. PLR and NLR of machine learning models for detecting LQTS (test/validation dataset)**


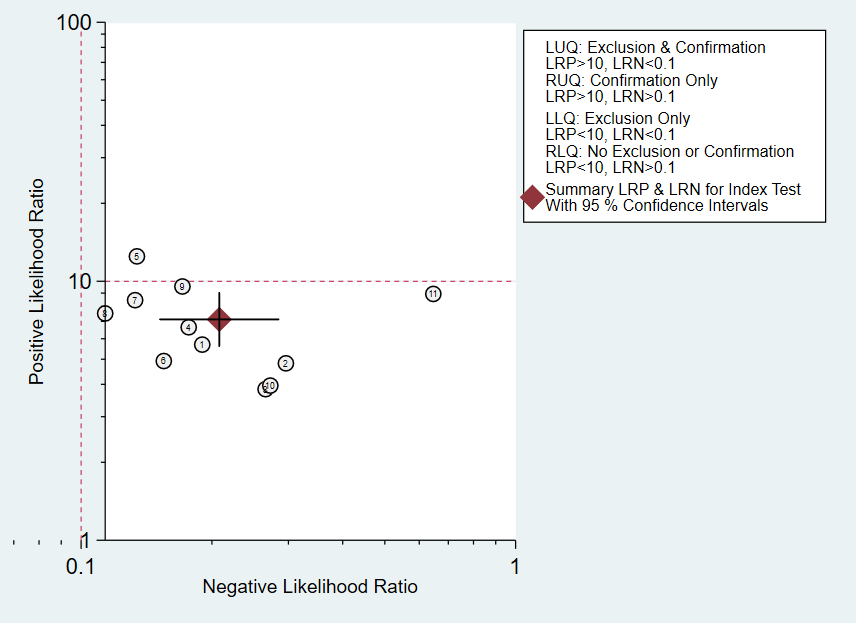


**Supplementary figure S17. Heterogeneity of machine learning models for detecting LQTS (test/validation dataset)**


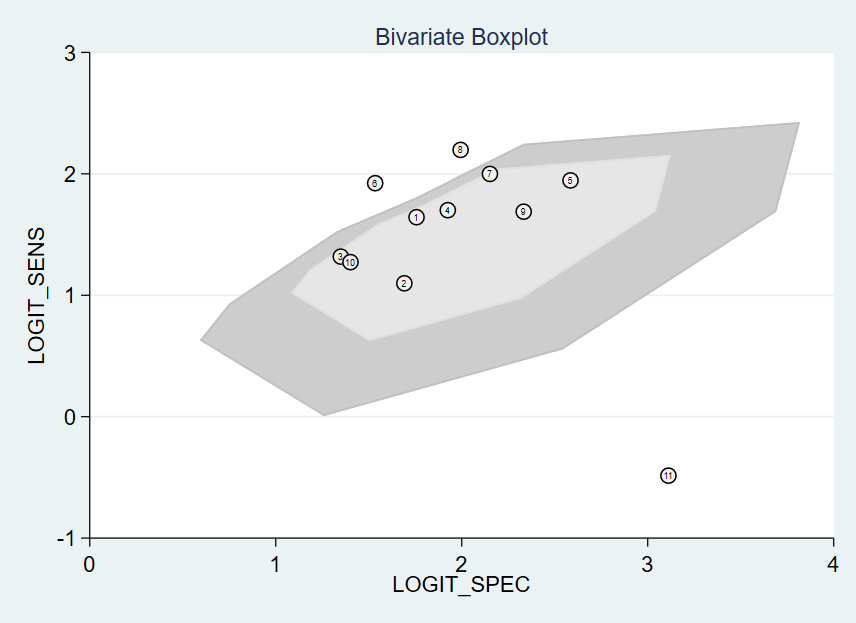

Supplement: Supplementary file 1 [file Table2.docx]
